# Sinomenine sensitizes gastric cancer cells to 5-fluorouracil *in vitro* and *in vivo*

**DOI:** 10.3892/ol.2013.1592

**Published:** 2013-09-18

**Authors:** FEI LIAO, ZIRONG YANG, XIAOHONG LU, XUFENG GUO, WEIGUO DONG

**Affiliations:** Department of Gastroenterology, Renmin Hospital of Wuhan University, Wuhan, Hubei 430060, P.R. China

**Keywords:** gastric carcinoma, 5-fluorouracil, sinomenine, thymidylate synthase, mitochondrial apoptosis

## Abstract

Sinomenine (SIN) has been reported to exert antitumor effects in various types of human cancer. The present study aimed to investigate the effects of SIN on gastric cancer and to briefly address its mechanism of action. In this study, the single and combined effects of SIN with 5-fluorouracil (5-FU) on human gastric cancer cells were assessed using an MTT assay, a combination index method and an MKN-28 xenograft mice model. Levels of apoptosis were determined using Hoechst 33258 staining and flow cytometry. Expression levels of certain apoptosis-related proteins were examined by western blotting. mRNA levels of the 5-FU-associated gene, thymidylate synthase (TS), were measured by RT-PCR. The results showed that SIN enhances 5-FU-mediated cellular growth inhibition and apoptosis in gastric cancer cells, reduces TS mRNA accumulation and activates the mitochondrial apoptotic pathway. The same chemotherapy sensitizer effect of SIN was confirmed *in vivo*. SIN is a promising chemotherapy sensitizer for 5-FU. Our results indicate that this may be a potential combination chemotherapeutic strategy for gastric cancer.

## Introduction

Gastric carcinoma is the fourth most common type of cancer worldwide and is the world’s second leading cause of cancer-related mortality (1). A combination of traditional treatments, such as curative surgery, radiotherapy and perioperative chemotherapy, may improve the survival rate of operable gastric carcinoma patients; up to 40–50% of patients who undergo potentially curative surgery alone ultimately relapse and die of metastatic disease (2). Therefore, surgery combined with chemotherapeutic agents may currently be the optimum strategy for gastric cancer therapy (3). Over the past 40 years, 5-fluorouracil (5-FU) has become the first-line chemotherapeutic agent for treating gastric carcinoma (4). However, low response rates and cell toxicity present obstacles to 5-FU-based chemotherapy (5,6). Thus, evaluation of the effect of new drugs or the effect of new combinations with established drugs is required. In addition, identification of novel agents that may be combined with 5-FU to achieve improved therapeutic effects and decreased host toxicity is a promising method.

Advances in the study of traditional Chinese medicine have led to the discovery of numerous novel chemotherapeutic agents. Sinomenine (7,8-didehydro-4-hydroxyl-3,7-dimethoxy-17-methylmorphinan-6-one; SIN), a bioactive alkaloid derived from a Chinese medicinal plant, has been demonstrated to be an effective therapy for rheumatic and arthritic diseases. Previous studies have demonstrated that the pharmacological profiles of SIN include immunosuppression, anti-inflammation and cytoprotection to exert anti-inflammatory and immunosuppressive activities (7,8). SIN has been reported to have an antitumor effect in several types of cancer cells, such as synovial sarcoma, lung cancer, hepatocellular carcinoma and gastric cancer (9–13). However, whether it sensitizes human gastric cancer cells to 5-FU has not yet been investigated. Thus far, there is little information available regarding the antitumor effects of SIN combined with 5-FU in human gastric cancer cells. The present study was designed to evaluate the efficacy of SIN when used in combination with 5-FU, and to explore the mechanisms underlying the effects of SIN and 5-FU. In this study, the *in vitro* inhibitory effects of SIN on the growth of several human gastric carcinoma cell lines were evaluated and cell apoptosis was detected *in vitro*. The *in vitro* inhibitory effect was verified using mouse xenograft models. The findings, particularly following *in vivo* verification, provide scientific evidence that a combination of SIN and 5-FU may be a promising anticancer therapeutic method, should the results be reproduced in clinical trials. The results of the present study may provide a novel perspective on gastric cancer therapy.

## Materials and methods

### Cell culture and reagents

Human gastric carcinoma cell lines, MKN-28, SGC-709, BGC-823 and HGC-27, were purchased from the Cell Bank of Type Culture Collection of Chinese Academy of Sciences (Shanghai, China). The cells were cultured in RPMI-1640 medium (Sigma-Aldrich, St. Louis, MO, USA), supplemented with 10% fetal bovine serum (Gibco-BRL, Gaithersburg, MD, USA), 50 mg/ml streptomycin, 50 IU/ml penicillin and 2 mM glutamine (Sigma-Aldrich), and the cell cultures were maintained in a 5% CO_2_ humidified atmosphere at 37°C. SIN and 5-FU were obtained from Sigma-Aldrich and dissolved in dimethylsulfoxide (DMSO; Sigma-Aldrich), and stock solutions (100 mM) were stored at −20°C.

### MTT assay and evaluation of the combined effects of SIN and 5-FU

Cells were seeded at a density of 4×10^3^ cells/well into a 96-well plate and allowed to attach overnight. The cells were treated with different drug groups (with or without the combination). For the control group, 0.1% DMSO was applied, which was the same concentration as that applied to the drug treatment groups. Upon termination of drug treatment, MTT (Sigma-Aldrich) was applied to each well at a final concentration of 0.5 g/l. Following incubation for 4 h at 37°C, the supernatant was discarded, 100 μl DMSO was applied and the MTT-formazan products were extracted. The absorbance was read at 570 nm using a 96-well microplate reader (Perkin-Elmer, Waltham, MA, USA). Each data point is the average of the results from five wells. Triplicate experiments with triplicate samples were performed. The results are expressed as inhibition rates (IRs), which were calculated using the following equation: IR = [(A−B)/A] × 100, where A and B represent the absorbance of the control and sample groups, respectively.

The combination index (CI) and isobologram methods of Chou and Talalay (14) and Chou *et al*(15) were used to evaluate the natural interaction between SIN and 5-FU. Assessment of the synergy, using a fixed constant ratio of the combination agents, was accomplished by calculating the CI and isobologram. The CI values were obtained using Biosoft CalcuSyn software (Biosoft, Cambridge, UK). CI<1, CI=1 and CI>1 indicate synergism, summation and antagonism, respectively.

### Detection of apoptotic cells by Hoechst 33258 staining and flow cytometry

The morphological features of apoptotic cells (chromatin condensation and fragmentation) were detected by Hoechst 33258 staining as follows: MKN-28 cells were treated with 100 mg/l 5-FU, 40 μM SIN or 50mg/l 5-FU + 20 μM SIN for 24 h, followed by incubation with 20 μM Hoechst 33258 (Sigma-Aldrich) for 10 min at room temperature. The cells were washed twice with phosphate-buffered saline (PBS) and examined under a Nikon 80i fluorescence microscope (Nikon Corporation, Tokyo, Japan). In each case, 10 random visual fields and >500 cells per field were counted.

The number of apoptotic cells was analyzed by flow cytometry using the MEBCYTO Apoptosis kit (MBL Co. Ltd., Nagoya, Japan). Briefly, 2×10^6^ cells were cultured in a 100-mm culture dish and harvested after a 24-h incubation period with 100 mg/l 5-FU, 40 μM SIN or 50 mg/l 5-FU + 20 μM SIN. The cells were then gently washed with PBS and resuspended in 100 μl of binding buffer. Annexin V-FITC (10 μl) and propidium iodide (5 μl) were applied to the resuspended cells. Following incubation at room temperature for 15 min in the dark, the stained cells were analyzed by flow cytometry using a single laser emitting excitation light at 488 nm (Bio-Rad Laboratories, Hercules, CA, USA).

### Western blot analysis

Cells were lysed in RIPA lysis buffer (Beyotime Institute of Biotechnology, Shanghai, China) for 20 min on ice, followed by centrifugation at 13,000 × g for 5 min. The extracted protein samples were separated on sodium dodecyl sulfate-polyacrylamide gel electrophoresis gels (40 μg/lane) and transferred to nitrocellulose membranes (Millipore, Bedford, MA, USA). The membranes were blocked with 5% skimmed milk in Tris-buffered saline and Tween 20 (TBST) buffer, and then incubated with primary antibodies overnight at 4°C. The primary antibodies and concentrations were as follows: Cytochrome *c* (1:500; Santa Cruz Biotechnology, Inc., Santa Cruz, CA, USA); β-actin (1:1,000; Santa Cruz Biotechnology, Inc.); and caspase-3 and caspase-9 (1:500; Cell Signaling Technology, Inc., Beverly, MA, USA). Following extensive rinsing with TBST buffer, the membranes were incubated with horseradish peroxidase-conjugated secondary antibodies (1:5,000; Pierce Biotechnology, Inc., Rockford, IL, USA). The bound antibodies were visualized using an enhanced chemiluminescence reagent (Amersham Pharmacia Biotech, Piscataway, NJ, USA) and quantified by densitometry using a Bio-Electrophoresis image analysis system (SF9-FR-980; Shanghai Furi Science and Technology Co., Ltd., Shanghai, China). Data are expressed as the relative density of the protein normalized to that of β-actin. The rates of inhibition were estimated by comparison with the untreated control (100%). Triplicate experiments with triplicate samples were performed.

### RT-PCR

Total RNA was extracted from the MKN-28 cells after a 24-h incubation period with 100 mg/l 5-FU, 40 μM SIN or 50 mg/l 5-FU + 20 μM SIN, using TRIzol^®^ reagent (Invitrogen Life Technologies, Carlsbad, CA, USA). Reverse transcription was performed using the First Strand cDNA Synthesis kit (Toyobo Co., Ltd., Osaka, Japan) according to the manufacturer’s instructions. Primer sequences were as follows: F: 5′-ACCAACCCTGACGACAGAAGA-3′ and R: 5′-AGCGC CATCAGAGGAAGATCT-3′ for thymidylate synthase (TS); and F: 5′-CCATCGTCCACCGCAAAT-3′ and R: 5′-TGCTC GCTCCAACCGACT-3′ for β-actin. β-actin was used as an internal control (housekeeping gene) in all experiments. PCR was performed using a Gene Cycler (Bio-Rad, Hercules, CA, USA). PCR products were confirmed by agarose gel electrophoresis. Gels were visualized and photographed under UV light, and the optical densities of the bands were analyzed using BandScan software, version 5.0 (Glyko, Inc., San Leandro, USA).

### Antitumor effects of SIN and 5-FU in vivo

Male outbred BALB/c-nu/nu mice (4 weeks of age) were purchased from the Animal Laboratory of Hubei Provincial Center of Disease Control (Wuhan, China), and maintained under specific pathogen-free conditions. The study was approved by the ethics committee of the Animal Care and Use Committee at Wuhan University (Wuhan, China). To establish human gastric xenografts, a density of 5.0×10^6^ MKN-28 cells in 0.2 ml PBS were inoculated into the lower right flank of each nude mouse (n=6 in each group) using a 24-gauge needle. Following growth for six days, the tumor xenografts reached a mean size of 100 mm^3^. Eighteen mice with tumor xenografts of ~100 mm^3^ in size were chosen and randomly divided into four groups: i) control (equal volume of physiological saline); ii) 40 mg/kg 5-FU; iii) 20 mg/kg SIN; and iv) 20 mg/kg 5-FU + 10 mg/kg SIN. All mice were administered the aforementioned drugs via intratumoral injection, once every three days. Following the last injection, all mice were sacrificed on day 30. During the autopsy procedure, the tumor was completely excised and weighed. Tumor diameters were measured at regular intervals with digital calipers, and the tumor volume in mm^3^ was calculated using the following formula: Volume = 0.5 × a × b^2^ (a, largest diameter; b, smallest diameter).

### TUNEL assay

For histological examination, tumor tissues were fixed in 10% buffered formalin and embedded in paraffin, and tissue sections (4-μm) were prepared. A TUNEL assay for apoptosis was conducted using an *In Situ* Cell Death Detection kit (Roche Diagnostics, Branchburg, NJ, USA) according to the manufacturer’s instructions. Positive cells were counted as the number of TUNEL-labeled cells per 100 epithelial cancer cells in 10 fields of the most affected tumor areas, with ×400 magnification, and analyzed using light microscopy (Carl Zeiss, Thornwood, NY, USA).

### Hematological side effects of SIN and 5-FU in vivo

To assess the hematological side effects of the chemotherapy *in vivo*, blood samples were collected before mice were sacrificed by cardiac puncture using heparin-rinsed 1-ml syringes (with 20-gauge needles; Shinva Medical Instrument Co., Ltd., Zibo, China), and were then centrifuged and maintained at 20°C until analyses. Standard techniques were adopted using an Olympus AU2700 analyzer (Olympus Optical Co., Ltd., Tokyo, Japan) to detect the activity of alanine aminotransferase (ALT), aspartate aminotransferase (AST), blood urea nitrogen (BUN) and serum creatinine (Cr); the biomarkers of liver and renal injury.

### Statistical analysis

Statistical analysis was performed using SPSS software, version 18.0 (SPSS Inc., Chicago, IL, USA). Data are expressed as the means ± SD, and comparisons between different groups were conducted by one-way analysis of variance. P<0.05 was considered to indicate a statistically significant difference.

## Results

### Growth inhibitory effect of SIN and/or 5-FU

**Figure 1:**
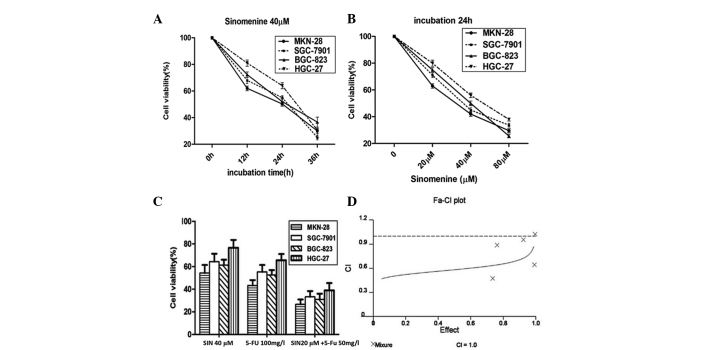
Evaluation of cell viability under the combined drug treatment. Cells were treated with (A) 40 μM SIN for 12, 24 or 36 h; (B) 20 μM, 40 μM or 80 μM SIN for 24 h; and (C) 40 μM SIN, 100 mg/l 5-FU or 20 μM SIN + 50 mg/l 5-FU for 24 h. (D) The effect of the combination therapy was assessed using a CI-isobologram. CI=1 indicates an additive effect, CI<1 indicates synergy between the two drugs and CI>1 indicates antagonism between the two drugs. Bars indicate the mean ± SD (n=3). SIN, sinomenine; 5-FU, 5-fluorouracil; DMSO, dimethylsulfoxide; CI, combination index.

The four gastric cancer cell lines were treated with SIN at various concentrations (20, 40 and 80 μM) for 24 h, or treated with 40 μM SIN for different time periods (12, 24 or 36 h) (Fig. 1A and B). As predicted, the cell viability was significantly inhibited by SIN treatment in a dose- and time-dependent manner.

Moreover, when half of the effective doses (20 μM SIN plus 50 mg/l 5-FU) of two drugs were combined together, the inhibitory effect was significantly higher than that of either of the full effective doses of drugs used individually (40 μM SIN or 100 mg/l 5-FU) (Fig. 1C).

The combinational inhibition rate was analyzed using the CI and isobologram methods of Chou and Talalay (14) and Chou *et al*(15). The experiments were repeated at least three times. The mean of the CI values was <1, indicating that SIN and 5-FU had a synergistic effect on proliferation inhibition of the gastric cancer cells (Fig. 1D).

### Apoptosis induced by SIN and 5-FU

**Figure 2:**
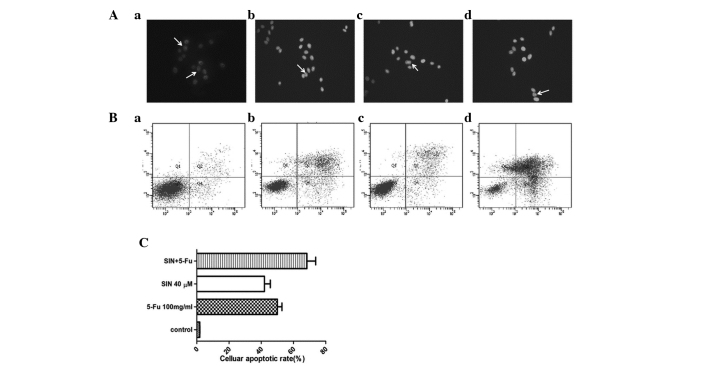
Cellular apoptosis in MKN-28 cells was analyzed by Hoechst 33258 staining and flow cytometry. (A) Apoptotic features were identified by observing chromatin condensation and fragmentation after Hoechst 33258 staining, as indicated by white arrows. (B) Detection of apoptosis via annexin V/PI staining (X-axis, annexin V; Y-axis, PI). The early apoptotic cells were defined as the sum of cells in the lower right quadrant of panel B. In A and B: (a) control; (b) 40μM SIN; (c) 100 mg/l 5-FU; and (d) 20 μM SIN + 50 mg/l 5-FU. (C) The apoptotic rate, as assessed via Hoechst 33258 staining. Bars indicate the mean ± SD (n=3). SIN, sinomenine; 5-FU, 5-fluorouracil; PI, propidium iodide.

The MKN-28 cells were exposed to 100 mg/l 5-FU, 40 μM SIN or 20 μM SIN + 50 mg/l 5-FU for 24 h. Morphological changes characteristic of apoptotic cells (chromatin condensation and fragmentation) were detected by Hoechst 33258 staining. Typical apoptotic nuclei are indicated by white arrows in Fig. 2A. The mean apoptotic rate in the SIN (40 μM), 5-FU (100 mg/l) and combination treatment (20 μM SIN + 50 mg/l 5-FU) groups were 40.37, 50.44 and 68.37%, respectively (Fig. 2C), demonstrating that SIN sensitized the gastric cancer cells to 5-FU-induced apoptosis. In addition, flow cytometry was performed to confirm that addition of SIN enhances 5-FU-induced early apoptosis (Fig. 2B and Table I).

**Figure 3:**
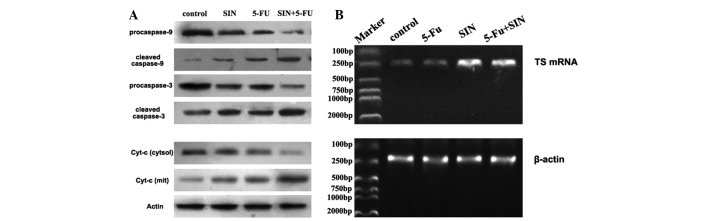
(A) Effect of 24 h of SIN and/or 5-FU treatment on the expression of apoptosis-related proteins in MKN-28 gastric cancer cells was assessed by immunoblotting analysis. Actin was used as an internal control. The lanes, from left to right, are as follows: Lane 1, control; lane 2, 40 μM SIN; lane 3, 100 mg/l 5-FU; and lane 4, 20 μM SIN + 50 mg/l 5-FU. (B) Effect of 24 h of SIN and/or 5-FU treatment on TS mRNA expression in MKN-28 cells. β-actin was used as an internal control. The lanes, from left to right, are as follows: Lane 1, marker; lane 2, control; lane 3, 100 mg/l 5-FU; lane 4, 40 μM SIN; and lane 5, 20 μM SIN + 50 mg/l 5-FU. SIN, sinomenine; 5-FU, 5-fluorouracil; Cyt-*c*, cytochrome *c*; TS, thymidylate synthase.

To further clarify the potential mechanisms by which SIN enhances 5-FU-induced apoptosis, the protein expression levels of cytochrome *c*, caspase-9 and caspase-3 were examined by western blot analysis. 5-FU treatment led to the release of cytochrome *c* from the mitochondria into the cytosol, and the activation of caspase-3 and caspase-9, and addition of SIN enhanced these changes (Fig. 3A).

### Expression of TS mRNA in 5-FU- and SIN-treated cells

To understand the molecular basis of the increased antitumor effects elicited by SIN, RT-PCR was performed to measure the expression of the 5-FU-associated gene TS, which is widely used to predict patients’ outcomes after chemotherapy. As shown in Fig. 3B, 5-FU treatment led to a decrease in the mRNA levels of TS in the MKN-28 cells, and SIN treatment potentiated this effect.

### Antitumor effects of SIN, 5-FU and combination treatment in vivo

**Figure 4:**
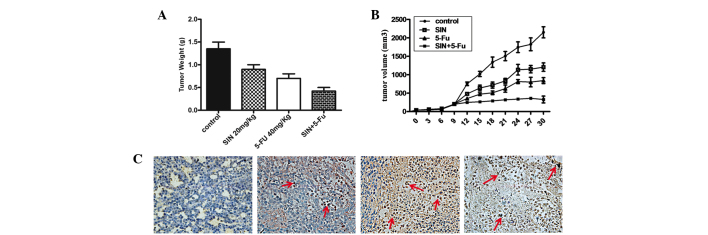
Antitumor effects of SIN and/or 5-FU *in vivo*. (A) Tumor weights were obtained at the end of the experiment. Error bars represent the SEM. P<0.05. (B) Tumor growth curve. Each time point represents the mean tumor volume for each group. Error bars represent the SEM. (C) Detection of apoptotic cells in tumor tissue by TUNEL staining: (a) Control group (equal volume of physiological saline vehicle); (b) 40mg/kg 5-FU group; (c) 20mg/kg SIN group; and (d) 20 mg/kg 5-FU + 10 mg/kg SIN group. All therapies were administered via intratumoral injection once every three days. Brown coloration indicating apoptotic signals is labeled with black arrows. Original magnification, ×400. Bars indicate the mean ± SD (n=3). SIN, sinomenine; 5-FU, 5-fluorouracil.

An *in vivo* study was also designed to evaluate the antitumor efficacy of SIN and/or 5-FU treatment in a gastric cancer xenograft model. Tumor volumes and weights were reduced sharply in the drug-treated group compared with those in the control group, though the degree of tumor suppression varied (Fig. 4A and B). The tumor volumes and weights of the combination group (20 mg/kg 5-FU + 10 mg/kg SIN) were lower than those of the SIN (20 mg/kg) and 5-FU (40 mg/kg) groups. These results demonstrate that the antitumor effect of SIN combined with 5-FU was superior to the effect of either drug used individually.

As previously described, SIN may render cells sensitive to 5-FU treatment by increasing the induction of apoptosis *in vitro.* To further examine this effect *in vivo*, an in situ TUNEL assay was used to detect apoptotic cells in subcutaneous tumor sections. The results demonstrate that apoptosis occurred in the SIN group, the 5-FU group and the combined group, whereas few apoptotic cells were found in the control group (Fig. 4C). Of the three drug-treated groups, the combined group exhibited a higher number of apoptotic bodies compared with that of the other two groups. The 10 mg/kg SIN combined with 20 mg/kg 5-FU treatment was generally well-tolerated by the mice during the long-term treatment.

### Evaluation of side effects in vivo

On completion of the experiment, the nude mice were sacrificed, and hepatic and renal toxicity were monitored by quantitative analysis of the serum ALT, AST, BUN and Cr levels. Notably, although the mice subjected to 5-FU showed increased levels of ALT, AST, BUN and Cr in the serum compared with those of the saline chloride control group (P>0.05), the addition of SIN did not induce any marked increases in the levels of ALT, AST, BUN and Cr in the serum (Table II). The blood cell count of the nude mice, including white blood count and platelet count were detected. The results indicated that SIN combined with 5-FU did not enhance the hematological side effects and no significant reduction in body weight was observed in the SIN or SIN + 5-FU groups (data not shown).

## Discussion

This study demonstrated that administration of SIN leads to an inhibitory effect on gastric cancer cells, and enhances the antitumor effects of 5-FU *in vitro* and *in vivo*. The key findings of this study include: i) SIN treatment may reduce cell viability and prominently increase tumor cell apoptosis; ii) addition of SIN may reduce the effective dose of 5-FU for gastric cancer treatment; iii) the inhibitory effect of 5-FU was notably elevated when combined with SIN, as evidenced by the detection of cell proliferation (tumor growth), apoptosis-related protein and the 5-FU-associated gene TS; and iv) the data obtained *in vivo* indicate that SIN has potential as a novel agent that sensitizes gastric cancer cells to 5-FU.

Gastric cancer usually has a poor prognosis and most patients are either diagnosed at an advanced stage or are subject to relapse following curative surgery (3,16). For advanced cancer patients, the currently available treatments are limited to systemic administration of conventional chemotherapy drugs, 5-FU and cisplatin, or their analogs, with or without an anthracycline. However, relying solely on these individual drugs does not improve the five-year survival rate of patients due to their severe side effects and associated drug resistance (17,18). Plant-derived compounds have attracted great interest due to their potential anticancer properties and low toxicity levels.

SIN is a bioactive alkaloid isolated from the Chinese herbal plant *Sinomenium acutum* Rehd. et Wils (Menispermaceae family). It has been utilized to treat rheumatic and arthritic diseases in China for >1,000 years (19,20). Increasing evidence has indicated that SIN exhibits antitumor actions in various types of cancer cells (9–12). However, its effect on gastric cancer remains unknown. The only study to date that has addressed the association between SIN and gastric cancer was that by Lv *et al*(13) in the USA. The authors indicated that SIN inhibits the proliferation of SGC-7901 gastric adenocarcinoma cells via suppression of cyclooxygenase-2 expression. Yet, whether SIN is able to sensitize gastric cancer cells to the effect of 5-FU is still not clear. The current study further confirmed that SIN inhibited the proliferation of several types of gastric cancer cells. It also demonstrated a synergistic antiproliferative effect of SIN with 5-FU, by inducing apoptosis in a time- and concentration-dependent manner.

Apoptosis is a highly regulated process that is activated by various stimuli that converge via different pathways. The mitochondrial pathway is considered to be pivotal in cell apoptosis. In the process, a number of stimuli cause the disruption of mitochondrial function and ultimately lead to the release of cytochrome *c* from the mitochondria into the cytosol (21). Cytochrome *c* then binds to Apaf-1, which further complexes with caspase-9 to form the apoptosome and promotes cleavage of downstream effector caspases (such as caspase-3) to trigger apoptosis (22–24). To elucidate the mechanisms underlying synergistic apoptosis induction by SIN and 5-FU, the present study investigated the expression of key apoptosis-related molecules. The data show that combining the 5-FU treatment with SIN increases cytochrome *c* release from the mitochondria into the cytosol, and increases the activation of caspase-3 and caspase-9, compared with that of 5-FU treatment alone. Therefore, our findings imply that the mitochondrial pathway is a key factor in enabling SIN to enhance 5-FU-induced apoptosis.

Another predominant finding of the present study was that SIN treatment significantly lowers the levels of TS mRNA. Previous studies have confirmed that TS is not only a key gene involved in 5-FU metabolism; it is closely associated with the resistance to 5-FU chemotherapy that is observed in numerous cancer patients. Three separate studies have identified that increased TS expression is clearly associated with resistance to 5-FU in murine colon adenocarcinoma and human gastrointestinal cancer cell lines (25–27). Conversely, several studies have revealed that decreased TS expression levels in tumors are closely associated with enhanced efficacy of 5-FU treatment (28–31). Consistent with these studies, the results of the present study showed that SIN treatment significantly inhibited TS mRNA expression; this effect may be responsible for SIN’s enhancement of sensitivity to 5-FU.

Collectively, the data presented in this study suggest that SIN may serve as a drug sensitizer for 5-FU in gastric cancer cells, and that the mechanisms underlying this effect may be associated with increases in apoptosis via the mitochondrial pathway and downregulation of TS mRNA expression. This indicated that a combination of SIN and 5-FU may result in an improved response to therapy in patients with gastric cancer compared with that in patients treated with 5-FU alone. These findings reveal a promising strategy to improve chemotherapeutic sensitivity in gastric cancer patients.

## Figures and Tables

**Table I:** Early cellular apoptotic rate (%).

Group	Early cellular apoptotic rate (%)
Control	1.1±0.09
SIN (40 μM)	15.2±1.35^a^
5-FU (100 mg/l)	20.8±17.20^a^
SIN (20 μM) + 5-FU (50 mg/l)	43.2±5.05^a^

Data are presented as the mean ± SD, n=3.

a P<0.05, versus the control.

SIN, sinomenine; 5-FU, 5-fluorouracil.

**Table II:** Analysis of the hematological index of SIN- and/or 5-FU-treated groups *in vivo*.

Group	n	ALT (U/l)	AST (U/l)	BUN (μmol/l)	Cr (μmol/l)	PLT (×10^9^/l)	WBC (×10^9^/l)
Control	6	37.50 (10.37)	125.00 (21.37)	7.23 (0.81)	17.27 (2.98)	105.7 (20.4)	7.3 (1.6)
SIN	6	42.33 (11.55)	135.83 (26.66)	8.02 (1.88)	20.26 (1.86)	103.9 (11.9)	7.6 (1.5)
5-FU	6	49.50 (16.50)	140.33 (42.65)	8.62 (1.18)	21.25 (3.00)	109.4 (18.0)	7.7 (2.0)
SIN+5-FU	6	39.00 (10.22)	131.17 (25.99)	7.42 (1.31)	19.89 (1.57)	110.5 (21.5)	7.9 (1.4)

Data are presented as the mean (SD), with n=6 mice/group. Mice in the different groups were treated as follows: Control (saline of equal volume); SIN (20 mg/kg); 5-FU (40 mg/kg); and SIN (10 mg/kg) + 5-FU (20 mg/kg). No statistically significant differences were observed in the ALT, AST, BUN, Cr, PLT and WBC levels among all groups. SIN, sinomenine; 5-FU, 5-fluorouracil; ALT, alanine aminotransferase; AST, aspartate aminotransferase; BUN, blood urea nitrogen; Cr, serum creatinine; PLT, platelet; WBC, white blood cells.

## References

[1] Jemal A, Bray F, Center MM, Ferlay J, Ward E, Forman D (2011). Global cancer statistics. CA Cancer J Clin.

[2] Jamal A, Murray T, Samuels A, Ghafoor A, Ward E, Thun M (2003). Cancer statistics, 2003. CA Cancer J Clin.

[3] Macdonald JS, Smalley SR, Benedetti J (2001). Chemoradiotherapy after surgery compared with surgery alone for adenocarcinoma of the stomach or gastroesophageal junction. N Engl J Med.

[4] Mackenzie M, Spithoff K, Jonker D (2011). Systemic therapy for advanced gastric cancer: a clinical practice guideline. Curr Oncol.

[5] Shekhar MP (2011). Drug resistance: challenges to effective therapy. Curr Cancer Drug Targets.

[6] Pasini F, Fraccon AP, DE Manzoni G (2011). The role of chemotherapy in metastatic gastric cancer. Anticancer Res.

[7] Qian L, Xu Z, Zhang W, Wilson B, Hong JS, Flood PM (2007). Sinomenine, a natural dextrorotatory morphinan analog, is anti-inflammatory and neuroprotective through inhibition of microglial NADPH oxidase. J Neuroinflammation.

[8] Wang Q, Li XK (2011). Immunosuppressive and anti-inflammatory activities of sinomenine. Int Immunopharmacol.

[9] Li X, Yue PY, Ha WY (2006). Effect of sinomenine on gene expression of the IL-1 beta-activated human synovial sarcoma. Life Sci.

[10] Jiang T, Zhou L, Zhang W, Qu D, Xu X, Yang Y, Li S (2010). Effects of sinomenine on proliferation and apoptosis in human lung cancer cell line NCI-H460 in vitro. Mol Med Report.

[11] Zhou L, Luan H, Liu Q, Jiang T, Liang H, Dong X, Shang H (2012). Activation of PI3K/Akt and ERK signaling pathways antagonized sinomenine-induced lung cancer cell apoptosis. Mol Med Report.

[12] Hong Y, Yang J, Shen X (2013). Sinomenine hydrochloride enhancement of the inhibitory effects of anti-transferrin receptor antibody-dependent on the COX-2 pathway in human hepatoma cells. Cancer Immunol Immunother.

[13] Lv Y, Li C, Li S, Hao Z (2011). Sinomenine inhibits proliferation of SGC-7901 gastric adenocarcinoma cells via suppression of cyclooxygenase-2 expression. Oncol Lett.

[14] Chou TC, Talalay P (1984). Quantitative analysis of dose-effect relationships: the combined effects of multiple drugs or enzyme inhibitors. Adv Enzyme Regul.

[15] Chou TC, Motzer RJ, Tong Y, Bosl GJ (1994). Computerized quantitation of synergism and antagonism of taxol, topotecan, and cisplatin against human teratocarcinoma cell growth: a rational approach to clinical protocol design. J Natl Cancer Inst.

[16] Dikken JL, van de Velde CJ, Coit DG, Shah MA, Verheij M, Cats A (2012). Treatment of resectable gastric cancer. Therap Adv Gastroenterol.

[17] Tsutani Y, Yoshida K, Sanada Y (2008). Decreased orotate phosphoribosyltransferase activity produces 5-fluorouracil resistance in a human gastric cancer cell line. Oncol Rep.

[18] Meyer HJ, Wilke H (2011). Treatment strategies in gastric cancer. Dtsch Arztebl Int.

[19] Liu L, Buchner E, Beitze D, Schmidt-Weber CB (1996). Amelioration of rat experimental arthritides by treatment with the alkaloid sinomenine. Int J Immunopharmacol.

[20] Gu B, Zeng Y, Yin C, Wang H, Yang X, Wang S, Ji X (2012). Sinomenine reduces iNOS expression via inhibiting the T-bet IFN-γ pathway in experimental autoimmune encephalomyelitis in rats. J Biomed Res.

[21] Boatright KM, Salvesen GS (2003). Mechanisms of caspase activation. Curr Opin Cell Biol.

[22] Li P, Nijhawan D, Budihardjo I, Srinivasula SM, Ahmad M, Alnemri ES, Wang X (1997). Cytochrome c and dATP-dependent formation of Apaf-1/caspase-9 complex initiates an apoptotic protease cascade. Cell.

[23] Yang J, Liu X, Bhalla K (1997). Prevention of apoptosis by Bcl-2: release of cytochrome c from mitochondria blocked. Science.

[24] Slee EA, Harte MT, Kluck RM (1999). Ordering the cytochrome c-initiated caspase cascade: hierarchical activation of caspases-2,-3,-6,-7,-8, and-10 in a caspase-9-dependent manner. J Cell Biol.

[25] Spears CP, Shahinian AH, Moran RG, Heidelberger C, Corbett TH (1982). In vivo kinetics of thymidylatesynthetase inhibition of 5-fluorouracil-sensitive and -resistant murine gastric adenocarcinomas. Cancer Res.

[26] Kitchens ME, Forsthoefel AM, Barbour KW, Spencer HT, Berger FG (1999). Mechanisms of acquired resistance to thymidylate synthase inhibitors: the role of enzyme stability. Mol Pharmacol.

[27] Kirihara Y, Yamamoto W, Toge T, Nishiyama M (1999). Dihydropyrimidine dehydrogenase, multidrug resistance-associated protein, and thymidylate synthase gene expression levels can predict 5-fluorouracil resistance in human gastrointestinal cancer cells. Int J Oncol.

[28] Peters G, Van der Wilt C, Van Triest B (1995). Thymidylate synthase and drug resistance. Eur J Cancer.

[29] Goekkurt E, Hoehn S, Wolschke C, Wittmer C, Stueber C, Hossfeld DK, Stoehlmacher J (2006). Polymorphisms of glutathione S-transferases (GST) and thymidylate synthase (TS) - novel predictors for response and survival in gastric cancer patients. Br J Cancer.

[30] Johnston PG, Lenz HJ, Leichman CG, Danenberg KD, Allegra CJ, Danenberg PV, Leichman L (1995). Thymidylate synthase gene and protein expression correlate and are associated with response to 5-fluorouracil in human colorectal and gastric tumors. Cancer Res.

[31] Lenz HJ, Leichman CG, Danenberg KD (1996). Thymidylate synthase mRNA level in adenocarcinoma of the stomach: a predictor for primary tumor response and overall survival. J Clin Oncol.

